# Prevalence of Insomnia in the Early Post-COVID-19 Recovery Period

**DOI:** 10.3390/ijerph192114224

**Published:** 2022-10-31

**Authors:** Robert Pudlo, Izabela Jaworska, Anna Szczegielniak, Jacek Niedziela, Zofia Kułaczkowska, Alicja Nowowiejska-Wiewióra, Jerzy Jaroszewicz, Mariusz Gąsior

**Affiliations:** 1 Department of Psychoprophylaxis, Faculty of Medical Sciences in Zabrze, Medical University of Silesia, 40-055 Katowice, Poland; 2 Department of Cardiac, Vascular and Endovascular Surgery and Transplantology, Faculty of Medical Sciences in Zabrze, Medical University of Silesia, 40-055 Katowice, Poland; 3 3rd Department of Cardiology, Silesian Center for Heart Disease, 41-800 Zabrze, Poland; 4 3rd Department of Cardiology, Faculty of Medical Sciences in Zabrze, Medical University of Silesia, 40-055 Katowice, Poland; 5 Department of Infectious Diseases and Hepatology, Faculty of Medical Sciences in Zabrze, Medical University of Silesia, 40-055 Katowice, Poland

**Keywords:** COVID-19, post-COVID-19 syndrome, insomnia, sleep disorders, Athens Insomnia Scale

## Abstract

Background: Sleep is a complex, reversible process that is responsible for the modulation of various physiological mechanisms. COVID-19-related sleep disorders are affecting different populations with a heterogenous prevalence, yet high rates among infected patients are frequently reported. The aim of the study is to assess the prevalence of insomnia in the early post-COVID-19 recovery period and explore the differences in the results acquired by the Athens Insomnia Scale (AIS) by gender and selected infection severity parameters. Methods: The data presented in the paper come from a prospective, observational study on COVID-19 complications (SILCOV-19) consisting of 200 COVID-19 patients. The AIS was used for the quantitative measurement of insomnia symptoms based on ICD-10 criteria. Results: 32% (*n* = 64) of all patients in the study group obtained results indicating sleep disturbances (>5 points on the scale), while 21.5% (*n* = 43) obtained results indicating insomnia (>10 points on the scale). The analysis of the results obtained by all patients in the AIS showed a significant correlation with the duration of symptoms (Spearman’s rank-order: R = 0.18; *p* < 0.05), but not with the number of days spent in the hospital or age. Women achieved a higher score in overall AIS, as well as in questions assessing total sleep time, well-being the next day, physical and mental fitness the next day, and sleepiness during the day (*p* < 0.05). Conclusions: the prevalence of insomnia in the early post-COVID-19 recovery period is high.

## 1. Introduction

Sleep is a complex, yet rapidly reversible process, consisting of different stages (wakefulness, light sleep, slow-wave sleep, and rapid eye movement), each characterized by a specific electrical pattern. It is responsible for the modulation of various physiological processes, being more than just a state of declined alertness, immobility, and decreased ability to respond to external stimuli [1]. It is vital to the proper functioning of the organism. Studies published so far indicate a two-way relationship: the disruption of the natural sleep pattern modifies various components of the immune system (including cytokine levels), while the immune response affects the sleep pattern [1]. There are reports showing that decreased total sleep time is correlated with prolonged suffering from infection [2]. Most infections, especially during the acute phase of the immune response, alter the sleep pattern by reducing wakefulness and the REM phase of sleep [3]. For example, the influenza virus, as based on laboratory animal studies, promotes NREM and declines the REM phase despite an observed decrease in body temperature [3]. However, viral infections are not homogeneous in terms of their impact on sleep. They can disturb its rhythm and/or promote/diminish its various phases [4]. The final clinical effect depends on both a direct and indirect influence over varied CNS structures and the functioning of other human body systems [4]. The direct influence is connected with neurotropism exhibited by some viruses and/or the immune response generated to fight the infection [4]. Penetration into the nerve and glial cells leads to damage to the structures of the nervous system and vascular endothelium [4]. The indirect influence may be connected with disturbances in the respiratory and endocrine systems [4]. Susceptibility and resistance to viral infections are also genetically determined [4]. These processes remain only partially investigated so far [4]. However, disrupted sleep in viral infections is linked with altered levels of proinflammatory cytokines and, consequently, is connected with the development of insomnia, agitation, and diminished sleep efficiency reported by patients [5]. Thus, the hypothesis on the trilateral relationship between the immune system’s aptitude, anxiety, and sleep efficiency has been recently presented [5]. Coronaviruses are found in the same group of sleep-influencing viruses as the human immunodeficiency virus (HIV), poliovirus, rabies virus, hepatitis viruses, and varicella-zoster virus. Disorders of the circadian rhythm may facilitate SARS-CoV-2 infection [6].

In the general population, several factors may play a role in the development of sleep dysfunctions related to the COVID-19 pandemic. So far age, gender, chronic diseases, psychosocial factors (including income, education, living conditions, media exposure) and higher consumption of alcohol are identified [7,8]. It is related both to the course of infection and to socioeconomic factors connected with changes in daily functioning in the epidemic era [7,8]. High rates of anxiety (ranging from 6.33% to 50.9%), depression (14.6–48.3%), post-traumatic stress disorder (7–53.8%), and psychological distress (34.43–38%) are also considered to have a reciprocal effect on sleep [9,10]. Insomnia, disrupted sleep continuity, changes in the circadian rhythm, excessive daytime sleepiness, sleep nightmares, restless leg syndrome, non-restorative sleep, and decreased sleep quality are only some of the symptoms of the wider phenomenon commonly named coronasomnia, or COVID-somnia [11]. It is considered a public health issue developing in parallel with the COVID-19 pandemic and resulting from a global lockdown that leads to social isolation, constant stress and anxiety [11]. COVID-19-related sleep disorders affect different populations, with a heterogenous prevalence of 30–40% in the general population [12]. Higher rates are reported among infected patients, reaching as many as 3/4 of all suffering from SARS-CoV-2 [12]. COVID-19 patients who experienced sleep disturbances had a higher incidence of hospital-acquired infection, longer hospitalization days, and an increased need for admission to ICU care than those without sleep disturbances [13]. Published studies so far suggest that the prevalence of sleep disorders is significantly higher in the post-COVID-19 group compared to the pre-COVID-19 group among those patients admitted to the intensive care unit or isolation unit. [14] While sleep disorders in patients can be attributed to the course of the acute phase of infection and side effects of medications, they are also present in the course of long-term COVID-19 [15]. The meta-analyses on the prevalence data showed that common residual symptoms among COVID-19 survivors at one-year post infection included fatigue/weakness (28%), dyspnoea (18%), arthromyalgia (26%), depression (23%), anxiety (22%), memory loss (19%), concentration difficulties (18%), and insomnia (12%) [15].

The aim of the study is to assess the prevalence of insomnia in the early post-COVID-19 recovery period and explore the differences in the results acquired in the Athens Insomnia Scale by gender and selected infection severity parameters.

## 2. Materials and Methods

Data presented in the paper come from a prospective, observational, registry-based Silesian study on COVID-19 complications (SILCOV-19) aimed at assessing post–COVID-19 complications in the Silesian population in Poland.

Inclusion criteria consisted of an age limit (>18 yo), well-defined diagnostic criteria in the acute phase of the infection, such as SARS-CoV-2 RNA confirmed by a polymerase chain reaction (PCR), and the presence of the clinical symptoms associated with COVID-19. In addition, 2 negative SARS-CoV-2 PCR test results following a 7-day period of quarantine after symptom regression were required. Lack of informed consent given by patients excluded from the study.

The enrolment began in June 2020 and finished in March 2021. Recruited COVID-19 patients were diagnosed in the Department of Infectious Diseases and Hepatology Clinic in Bytom, divided into 2 groups according to their hospitalization status, and appointed for a study visit at the Silesian Center for Heart Disease in Zabrze. The study visits were scheduled after at least 60 days from the first symptom(s), and for the purposes of the work, a definition of the post-COVID-19 syndrome was adopted as “persistent symptoms and/or delayed or long-term complications of SARS-CoV-2 infection beyond 4 weeks from the onset of symptoms that cannot be explained by an alternative diagnosis” [16]. The multidisciplinary research included complex cardiovascular, pulmonary, neurological, and hepatological diagnostics with laboratory (30 different parameters from blood samples), imaging (chest X-ray and high-resolution computer tomography), and functional tests (6 MWT). All of the results were consulted with a neurologist and vascular surgeon. Nineteen most common and well-documented symptoms related to COVID-19 in the acute phase, after 1 week, after 1 month and at the time of testing were assessed. Additionally, mental health after COVID-19 was investigated; the assessment by selected clinical tools (Athens Insomnia Scale, Hospital Anxiety and Depression Scale, State-Trait Anxiety Inventory, Ergo-Resiliency Assessment) was performed for each patient by the same member of the research team to avoid any bias. There was no separate scale dedicated to the severity of COVID-19.

The detailed methodology, baseline characteristics of the study group, and general results of the Silesian Study on COVID-19 complications in hospitalized and non-hospitalized patients have been published in a separate article [17].

The Athens Insomnia Scale (AIS) consists of 8 test items assessing: falling asleep, waking up at night, waking up in the morning, total sleep time, sleep quality, well-being the next day, mental and physical performance the next day, and sleepiness during the day. It allows quantitative measurement of insomnia symptoms based on the ICD-10 criteria. The total score of 6 points or more is considered a high probability of insomnia presence. The scale has been validated in Polish conditions [18].

The study was approved by the Bioethical Committee of the Medical University of Silesia in Katowice (17/2020, 1 June 2020) and registered at ClinicalTrials.gov (NCT04 453 748, https://clinicaltrials.gov/ct2/show/NCT04453748 (accessed on 2 August 2022)).

The collected data were processed via the Statistica 13.3 program, licensed by the Medical University of Silesia in Katowice. A Shapiro–Wilk test was used to assess the normality of distributions. The Mann–Whitney U test for a dichotomous grouping variable was used to compare quantitative variables. The relationships between quantitative variables were assessed using the Spearman’s rank correlation coefficient. Statistical significance was defined as *p* <⁠ 0.05.

## 3. Results

The study group consisted of 200 COVID-19 patients (101 men, 50.5% vs. 99 women, 49.5%) of which 113 patients were not hospitalized due to the infection (50 men, 44.2% vs. 63 women, 55.8%). The distribution of the following parameters: age [years], duration of symptoms [days], hospitalization [yes/no], and the number of days in the hospital by gender is presented in the table below (Table 1).

**Figure 1:**
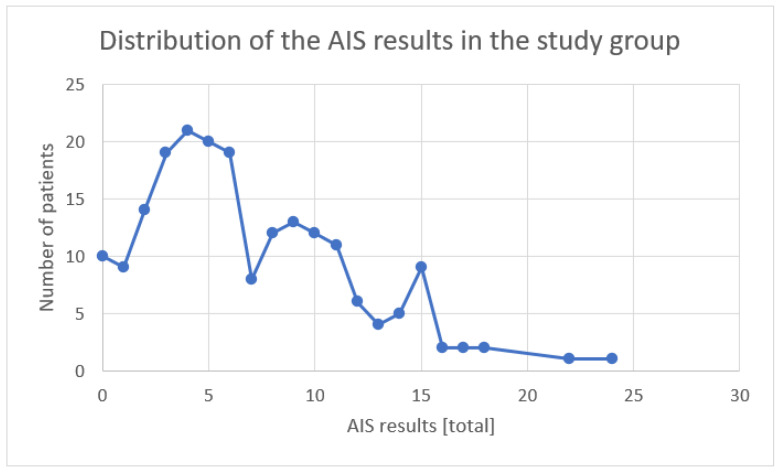
Distribution of the AIS results in the study group.

In the study group of patients who contracted COVID-19 of both genders and completed fully the AIS (*n* = 200), the results presented themselves as follows: 46.5% (*n* = 93) of the patients did not show any sleep disturbances, and 32% (*n* = 64) of the patients had a result indicating sleep disturbances (>5 points on the scale), while 21.5% (*n* = 43) of the patients had a result indicating insomnia (>10 points on the scale) (Figure 1).

The analysis of the results obtained by all of the patients in the AIS shows a significant correlation with the duration of symptoms (days) (Spearman’s rank-order: R = 0.18; *p* < 0.05), while the number of days spent in the hospital and the age did not correlate with the overall result obtained on the scale. In the individual aspects assessed in the AIS, the duration of COVID-19 symptoms correlated with falling asleep, waking up at night, waking up early, feeling well the next day, and with psychophysical fitness the next day (Spearman’s rank-order: R = 0.23, R = 0.19, R = 0.19, R = 0.22, R = 0.15, and R = 0.18; *p* < 0.05). The number of days spent in the hospital correlated with falling asleep and daytime sleepiness (Spearman’s rank-order: R = 0.15 and −R = −0.25; *p* < 0.05). Age correlated only with falling asleep and waking up at night (Spearman’s rank-order: R = 0.20 and R = 0.17; *p* < 0.05).

Among the women who contracted COVID-19 and completed fully the AIS (n = 99), the results presented themselves as follows: 39.4% (n = 39) of the patients did not show any sleep disturbances, and 32.3% (n = 32) of the patients had a result indicating sleep disturbances (>5 points on the scale), while 28.3% (n = 28) of the patients had a result indicating insomnia (>10 points on the scale).

Among the men who contracted COVID-19 and completed fully the AIS (n = 101), the results presented themselves as follows: 53.5% (n = 54) of the patients did not show any sleep disturbances, and 31.7% (n = 32) of the patients had a result indicating sleep disturbances (>5 points on the scale), while 14.8% (n = 15) of the patients had a result indicating insomnia (>10 points on the scale).

**Figure 2:**
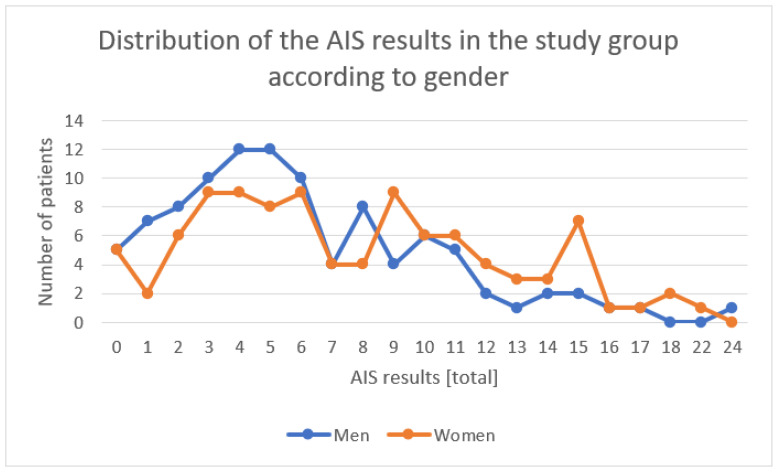
Distribution of the AIS results in the study group according to gender.

The descriptive statistics for all of the AIS questions, separately, and the distribution of the overall results are presented below for the general study group and divided by gender (Table 2, Figure 2).

**Figure 3:**
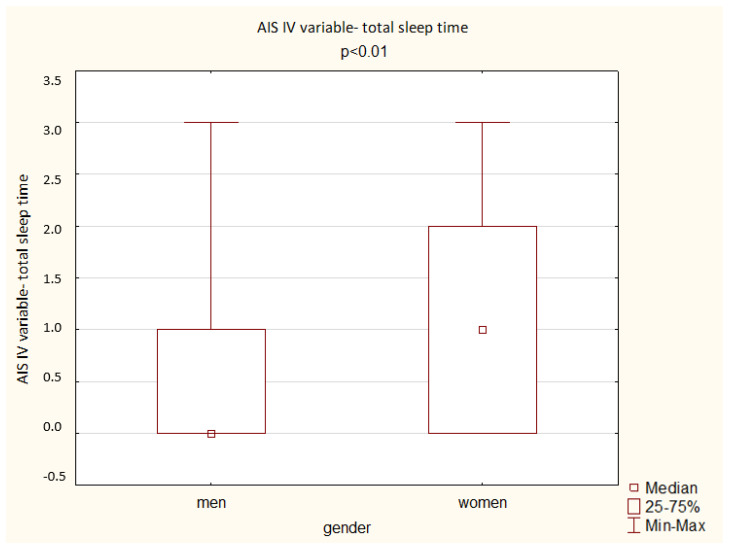
Patient results in the AIS IV variable—total sleep time (Mann–Whitney U test).

**Figure 4:**
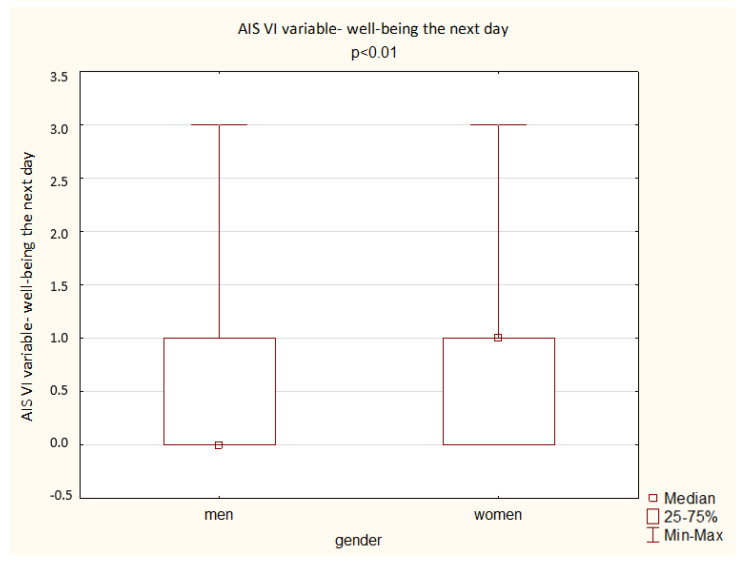
Patient results in the AIS VI variable—well-being the next day (Mann–Whitney U test).

**Figure 5:**
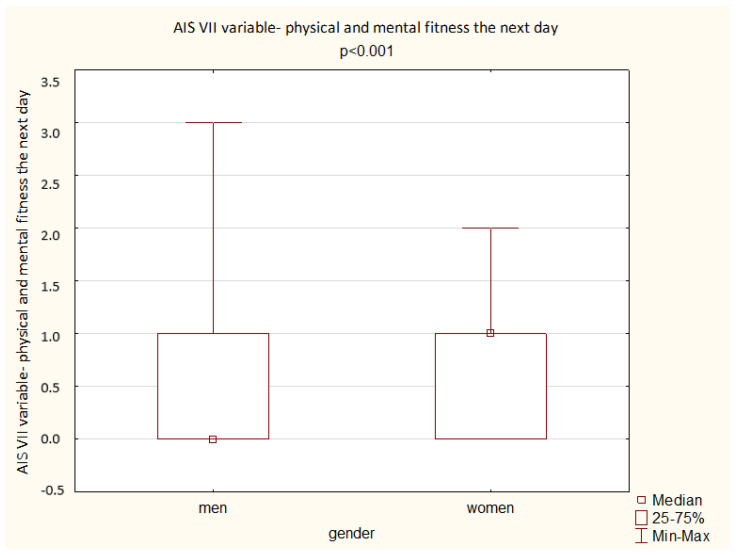
Patient results in the AIS VII variable—physical and mental fitness the next day (Mann–Whitney U test).

**Figure 6:**
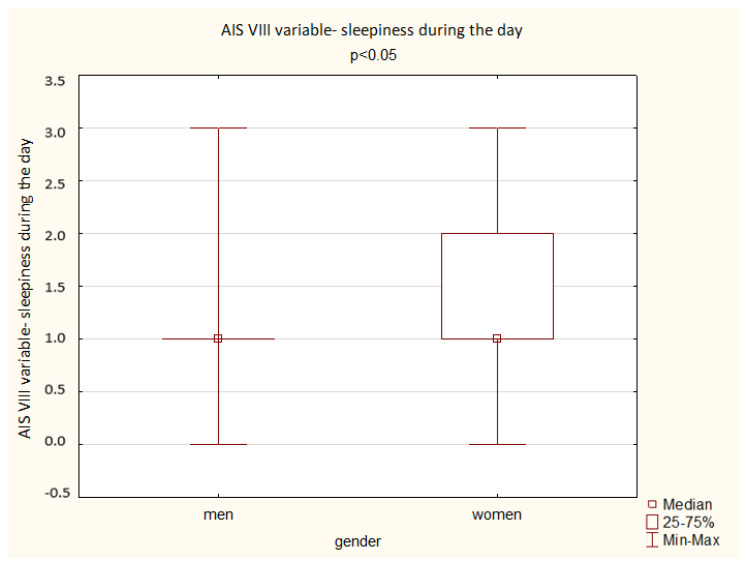
Patient results in the AIS VIII variable—sleepiness during the day (Mann–Whitney U test).

**Figure 7:**
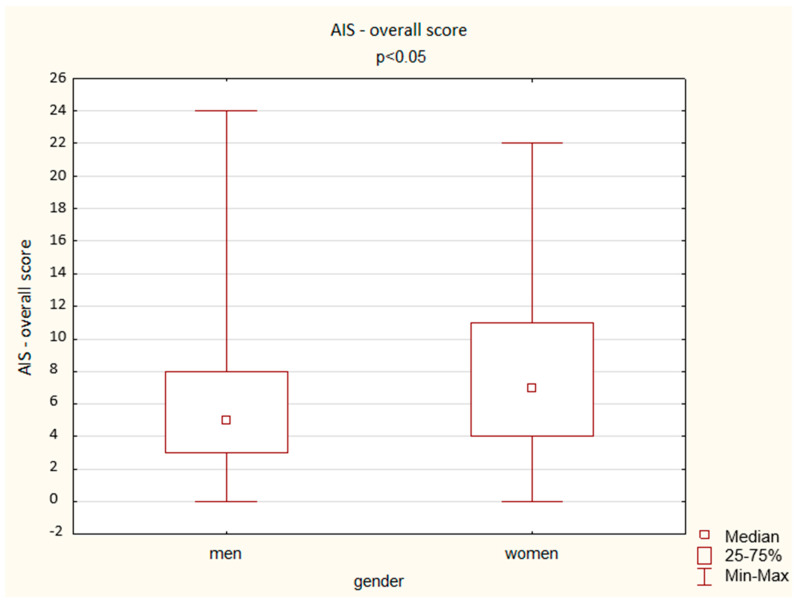
Patient results in the AIS—overall score (Mann–Whitney U test).

A comparison of the results obtained by the men and women in the AIS shows a significant difference depending on gender: women achieved a higher overall score but also higher scores in the questions assessing total sleep time, well-being the next day, physical and mental fitness the next day, and sleepiness during the day (*p* < 0.05). The results are presented below (Figure 3, Figure 4, Figure 5, Figure 6 and Figure 7).

Among the hospitalized patients due to contracted COVID-19 who completed fully the AIS (n = 86), the results presented themselves as follows: 45.3% (n = 39) of the patients did not show any sleep disturbances, and 27.9% (n = 24) of the patients had a result indicating sleep disturbances (>5 points on the scale), while 26.7% (n = 23) of the patients had a result indicating insomnia (>10 points on the scale).

**Figure 8:**
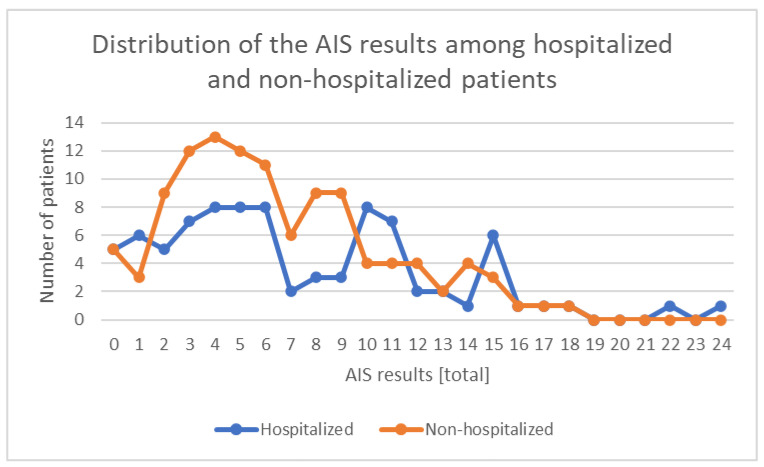
Distribution of the AIS results among hospitalized and non-hospitalized patients.

Among the non-hospitalized patients who contracted COVID-19 and completed fully the AIS (n = 113), the results presented themselves as follows: 47.8 (n = 54) of the patients did not show any sleep disturbances, and 34.5% (n = 39) of the patients had a result indicating sleep disturbances (>5 points on the scale), while 17.7% (n = 20) of the patients had a result indicating insomnia (>10 points on the scale). The distribution of the AIS overall results among hospitalized and non-hospitalized patients is presented below (Figure 8).

The descriptive statistics divided between hospitalized and non-hospitalized patients for all of the AIS questions are presented below (Table 3).

**Figure 9:**
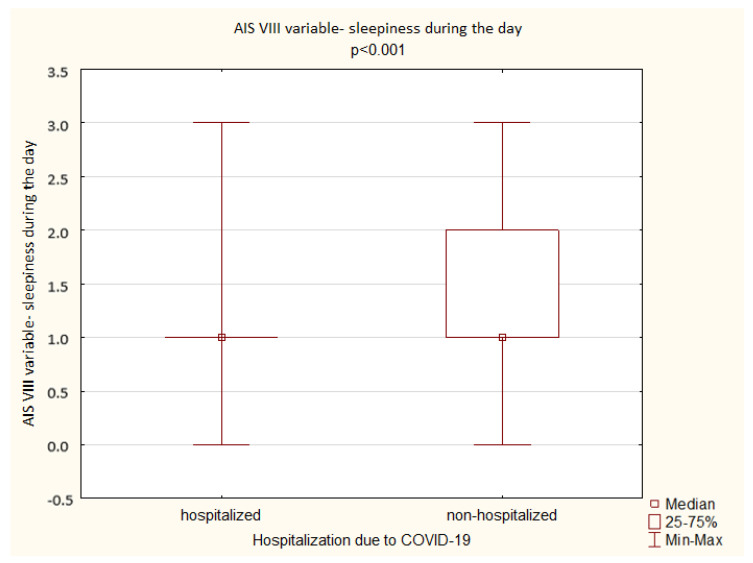
Hospitalized vs. non-hospitalized patients’ results in the AIS VIII variable—sleepiness during the day (Mann–Whitney U test).

A comparison of the results obtained by the hospitalized and non-hospitalized patients in the AIS shows no significant difference in the overall AIS score as well as in the individual scale aspects with an exception of sleepiness during the day (*p* < 0.001) (Figure 9).

**Figure 10:**
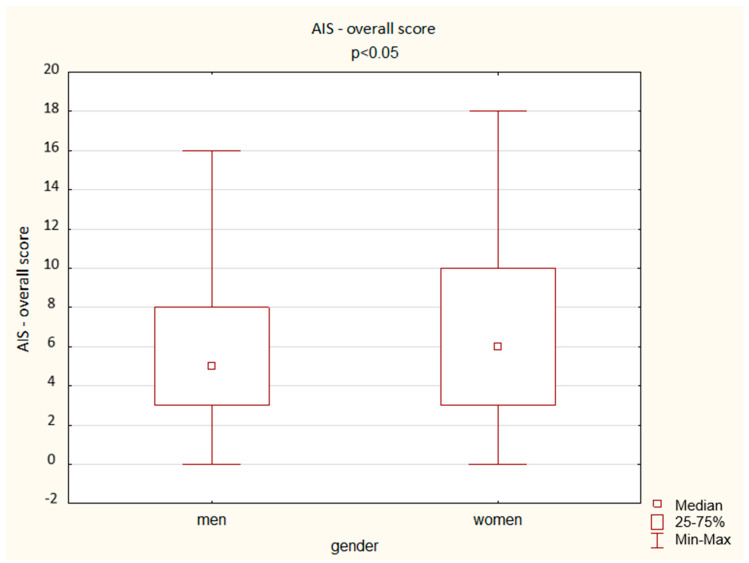
Hospitalized patient results in the AIS—overall score (Mann–Whitney U test).

A comparison of the results obtained by the hospitalized men and women in the AIS shows a significant difference depending on gender: women achieved a higher overall score (*p* < 0.05) but also higher scores in the questions assessing waking up at night (*p* < 0.05), total sleep time (*p* < 0.01), well-being the next day (*p* < 0.01), physical and mental fitness the next day (*p* < 0.05), and sleepiness during the day (*p* < 0.001) (Figure 10).

**Figure 11:**
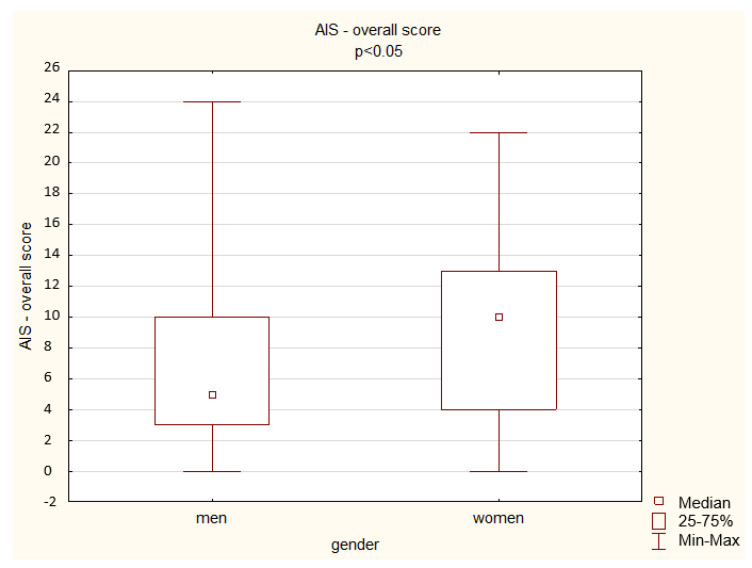
Non-hospitalized patient results in the AIS—overall score (Mann–Whitney U test).

A comparison of the results obtained by the non-hospitalized men and women in the AIS shows a significant difference depending on gender only in one aspect of the questionnaire: women achieved a higher score in total sleep time (*p* < 0.01) (Figure 11).

The descriptive statistics for all of the AIS questions, separately, and the overall results are presented below for both hospitalized and non-hospitalized patients divided by gender (Table 4).

While hospitalization is a direct parameter stating the severity of the infection, an additional correlation of the results obtained in the AIS with the number of symptoms related to COVID-19 (fever, fatigue, anorexia, muscle pain, cough, headache, body weight loss of ≥2 kg, ageusia, anosmia, diarrhea, abdominal pain, dyspnea, sore throat, chest pain, vomiting, skin diseases, hair loss, palpitations, and leg edema) in the acute phase, after 1 week, after 1 month, and at present was performed. The difference between the number of symptoms in the acute phase and now was also assessed. The descriptive statistics for the number of symptoms related to COVID-19 by different times of the assessment are presented below (Table 5).

A comparison of the number of symptoms related to COVID-19 presented by men and women shows a significant difference depending on gender only in regards to acute phase and the difference between the number of symptoms in the acute phase and now (*p* < 0.05; Mann–Whitney U test). The same analysis for hospitalized and non-hospitalized patients shows a significant difference only in regards to the difference between the number of symptoms in the acute phase and now (*p* < 0.05; Mann–Whitney U test).

For the study group, the significant yet low correlation between the results of the AIS and the number of symptoms at present was identified in regards to: falling asleep, waking up at night, waking up in the morning, total sleep time, sleep quality, well-being the next day, mental and physical performance the next day, and the total score of the AIS (Spearman’s rank-order: R = 0.30, R = 0.25, R = 0.16, R = 0.21, R = 0.22, R = 0.24, R = 0.21, and R = 0.27; *p* < 0.05). The same correlation, but for the acute phase’s symptoms, was significant for total sleep time, well-being the next day, and mental and physical performance the next day (Spearman’s rank-order: R = 0.18, R = 0.21, and R = 0.23; *p* < 0.05); for the symptoms present after 1 month, the correlation was significant for waking up at night and waking up in the morning (Spearman’s rank-order: R = 0.18 and R = 0.17; *p* < 0.05); for the difference between the number of symptoms in the acute phase and now, the correlation was significant for all of them except waking up in the morning and sleepiness during the day.

For the men in the study group, the significant yet low correlation between the results of the AIS and the number of symptoms at present was identified in regards to: waking up at night, total sleep time, sleep quality, and the total score of the AIS (Spearman’s rank-order: R = 0.23, R = 0.19, R = 0.23, and R = 0.20; *p* < 0.05). The same correlation, but for the acute phase’s symptoms, was significant for well-being the next day and mental and physical performance the next day (Spearman’s rank-order: R = 0.20 and R = 0.21; *p* < 0.05); for the difference between the number of symptoms in the acute phase and now, the correlation was significant for total sleep time, sleep quality, well-being the next day, mental and physical performance the next day, and the total score of the AIS (Spearman’s rank-order: R = 0.23, R = 0.22, R = 0.25, R = 0.27, and R = 0.24; *p* < 0.05).

For the women in the study group, a significant correlation between the results of the AIS and the number of symptoms at present was found in regards to all of the parameters except sleepiness during the day and the total score of the AIS (Spearman’s rank-order: R = 0.46, R = 0.27, R = 0.20, R = 0.23, R = 0.21, R = 0.30, and R = 0.31; *p* < 0.05). The same correlation, but for the acute phase’s symptoms, was significant only for mental and physical performance the next day (Spearman’s rank-order: R = 0.20 and R = 0.21; *p* < 0.05); for the symptoms present after 1 month, the correlation was significant for waking up at night, waking up in the morning, sleep quality, and well-being the next day (Spearman’s rank-order: R = 0.24, R = 0.24, R = 0.25, and R = 0.21; *p* < 0.05); for the difference between the number of symptoms in the acute phase and now, the correlation was significant for waking up at night, total sleep time, sleep quality, well-being the next day, and mental and physical performance the next day (Spearman’s rank-order: R = 0.29, R = 0.20, R = 0.21, R = 0.33, and R = 0.30; *p* < 0.05) (Table 6).

For the hospitalized patients, the significant yet low correlation between the results of the AIS and the number of symptoms at present was identified only in regards to waking up at night and well-being the next day (Spearman’s rank-order: R = 0.25 and R = 0.21; *p* < 0.05). The same correlation, but for the acute phase’s symptoms, was significant only for well-being the next day and mental and physical performance the next day (Spearman’s rank-order: R = 0.24; *p* < 0.05.

For the non-hospitalized patients, the significant yet low correlation between the results of the AIS and the number of symptoms at present was identified in regards to all of the parameters except sleepiness during the day (Spearman’s rank-order: R = 0.37, R = 0.22, R = 0.24, R = 0.30, R = 0.27, R = 0.26, R = 0.27, and R = 0.34; *p* < 0.05). The same correlation, but for the acute phase’s symptoms, was significant for total sleep time, well-being the next day, mental and physical performance the next day, and sleepiness during the day (Spearman’s rank-order: R = 0.20, R = 0.23, R = 0.22 and R = 0.22; *p* < 0.05) (Table 7).

## 4. Discussion

Insomnia has been named one of the most common neurological and psychiatric outcomes among COVID-19 survivors. The results from an analysis of electronic health records of 236,000 COVID-19 patients show that 5.4% experienced insomnia [19]. The number only increases with infection severity and a need for hospitalization [19]. The same study indicated that insomnia was also more common in COVID-19 survivors than in those who had influenza or other respiratory tract infections [19]. A paper on sleep disorders among French COVID-19 survivors (n = 106) reported 43.3% of clinical insomnia cases with 35.8% of moderate and 7.5% of severe course in a month’s time after hospital discharge [20]. Korean studies based on data from the National Health Insurance Service presented a 3.33-fold higher prevalence of insomnia among COVID-19 survivors with 5.4% of them newly diagnosed with an insomnia disorder at the 6-month follow-up. At the same time, researchers suggested that the severity of symptoms does not correlate with the presence of insomnia and patients without symptoms present a higher prevalence of sleep disorders in comparison to the control group [21]. In our study, around half of the COVID-19 survivors achieved an overall AIS score that excludes the diagnosis of insomnia; however, there was no significant difference between the hospitalized and non-hospitalized patients. It seems to be in line with studies published so far in terms of both the prevalence of insomnia and the direct influence of the coronavirus on sleep. The neurotropism of the virus described in publications and the consequent damage of the CNS from the structural and functional point of view may explain the obtained results. Dynamic changes characterizing neuroinflammation had been connected with sleep loss and the subsequent onset of depression. Women and young adults have been identified to present more potent inflammation, which is also in line with the epidemiologic results pointing to these groups. They seem to be at greater risk of depression as sleep disturbance may serve as a vulnerability factor to increase the severity of depressive symptoms [22]. This may also explain why the results obtained do not confirm a correlation between age and insomnia, despite the physiological aging process that may lead to a deterioration in sleep quality and changes in sleep patterns [23]. The negative correlation between the length of hospitalization and lower daytime sleepiness can be explained by the effectiveness of the treatment.

A recently published study indicates that the global rate of sleep problems during COVID-19 epidemic is approx. 35.7%, with higher rates for healthcare workers (36.0%) and lower for the general population (32.3%). It also points out that COVID-19 patients present a higher rate of sleep disturbances (74.8%) [24]. In the metanalysis from 2022 the prevalence of insomnia symptoms in different populations was assessed: university students (58.4%), infected patients in the acute phase of COVID-19 (54.1%), and pregnant women (53.3%) reported highest rates of insomnia during the epidemic. They were followed by COVID-19 survivors (40.1%) and health workers (39.3%), while the general population presented the lowest rates of symptoms (29.7%). The same study showed that people with a history of mental disorders displayed over three times higher risk for depression/anxiety and nearly two times higher risk for insomnia [25]. The data concerning the population of students in Poland during COVID-19 showed that more than 50% of them had some form of sleep disturbances with moderate-to-severe insomnia symptoms noted in 21.6% [26]. Another multi-centered study from 13 countries seems to confirm a higher risk of insomnia for COVID-19 survivors, but also points out additional socioeconomic factors negatively affecting the sleep, such as financial burden, isolation for a period of four to five weeks, and living alone or with more than five people in the same household [27]. An additional element that introduces difficulties in evaluating the prevalence of the phenomenon and identifying the risk factors for its development is that studies assess the patients at different times after COVID-19 infection. The knowledge we currently have at our disposal is also significantly influenced by the variety of diagnostic tools used by researchers. Studies based on the analysis of the electronic data of healthcare systems are also limited by the diagnoses and classifications of mental disorders used.

Some papers have reported that female gender and self-perceived illness severity were significantly associated with symptoms of depression, anxiety, and insomnia of psychosocial factors may play role in the process [27]. The studies published so far do not allow us to make an unambiguous assessment in terms of significant gender differences in the prevalence of sleep disorders during the COVID-19 epidemic, while at the same time attention is being paid to gender-specific estimates of sleep problems [28,29]. The results of our study suggest a higher risk of insomnia among women. With regards to the Polish population in the pandemic, studies evaluating the outcome of the Athens Insomnia Scale showed an increase in insomnia rates for both psychiatric patients and control groups from the general population. Greater symptoms are associated with the female gender as compared to the male gender. According to the authors of the study, the identified gender difference is in line with the occurrence of insomnia in the general population in Poland, where sleep disorders are about 1.5x more common among women [30]. Research on Mexican patients with mild, moderate, and severe symptoms of COVID-19 suggests that pre-existing heart disease, anxiety, and cognitive complaints before the infection may be risk factors for developing insomnia [31]. The identification of somatic factors seems to be particularly important and indicates a multifactorial process disrupting the quality and structure of sleep in COVID-19 patients. Interestingly, in our studies, the differences between both genders were more visible with higher severity of symptoms when the hospitalized and non-hospitalized groups were compared. It should be considered whether the severity of the course of acute infection is actually a parameter that allows us to predict the occurrence of mental health and sleep symptoms in the selected population, or whether the observation of the course of long-COVID-19 is a more important aspect. A recently published work describes post-COVID profiles and how they relate to different viral variants and vaccination status; researchers identified three clusters of symptoms associated with different SARS-CoV-2 variants (cardiorespiratory, neurological, and multi-organ/gastrointestinal) [32]. This may also explain the presented differences in the results obtained in the AIS between the hospitalized and non-hospitalized patients in relation to the number of persistent COVID-19 symptoms.

Before the pandemic, population-based studies have shown that psychiatric disorders and sleep disturbances are frequently reported together with a bidirectional character of the relationship between them (a strong correlation is proven, among others, with a depressive disorder, generalized anxiety disorder, and alcohol abuse) [33,34]. Insomnia was present in 19.7% of the students attending seven different universities in Poland, and its presence correlated with the intensity of perceived stress [35]. The study from 2022 indicates that the COVID-19 pandemic, through loneliness, anxiety, fear, stress, extreme tiredness, social isolation, juggling work or study, parenting challenges, significant behavior changes, and a variety of health concerns, is connected with widely spread mental health challenges. Individual and social hardships with financial insecurity have led to the disruption of circadian rhythms, affecting all sleep regulatory processes (homeostatic sleep drive, the circadian rhythm, and the arousal system) [36]. Thus, an assessment of insomnia and different sleep disorders requires an in-depth assessment of mental state.

The presented work has its limitations. First of all, research based on questionnaires always hold a certain bias. Secondly, the lack of a generally accepted definition and time frame for the long-term COVID-19 assessment makes it impossible to compare the obtained results in a wider perspective. The characterization of the early recovery period based on a resolution of the acute symptoms, as well as the level of severity of the reported symptoms, seems, as well, to vary in terms of the duration and listed signs depending on the authors’ assumptions and methodologies of the presented research. Thirdly, the sleep-related symptoms were based on the respondents’ self-reports than clinical diagnoses. The heterogeneity of the published data so far suggests the need to assess a wider range of risk factors related to mental health, somatic health, and socioeconomic conditions when discussing COVID-19-related insomnia both in the general population and among COVID-19 survivors.

## 5. Conclusions

1. The prevalence of insomnia in the early post-COVID-19 recovery period is high, with one out of five survivors meeting the clinical criteria of the diagnosis;
2. The duration of COVID-19 symptoms correlates positively with the development of insomnia;
3. The number of persisting COVID-19 symptoms positively correlates to a greater degree with insomnia than the number of symptoms observed in the acute phase of the disease;
4. Significant gender differences in reported insomnia have been shown, but the exact relationships require further research.

## 6. Suggestions on the Way Forward

Both our research and other publications on insomnia in the pandemic era indicate that it is a significant problem affecting the quality of life and prognosis in comorbidities. Since no standards for the treatment of insomnia specific for people with COVID-19 and convalescent patients have been developed so far, standard methods of treatment should be used, with the greatest attention being paid to the early implementation of pharmacological treatment in post-COVID insomnia. Taking into account the chronicity of insomnia in patients with COVID-19 and the frequent coexistence of insomnia and depression in this population, it seems rational to avoid drugs with an addictive potential and to conduct treatment primarily with antidepressants with a hypnotic potential.

## Figures and Tables

**Table 1:** Characteristics of the assessed parameters by gender.

	Median [Men]	Median [Women]	Min [Men]	Min [Women]	Max [Men]	Max [Women]	Valid N [Men]	Valid N [Women]	SD [Men]	SD [Women]	*p* Value	F Variances	*p* Value Variances
AIS [total]	6.09901	7.70707	0	0	24	22	101	99	4.3371	4.87026	0.01447	1.260995	0.24991
Duration of symptoms [days]	10.51485	11.92857	0	0	21	31	101	99	4.6554	5.9815	0.063842	1.650881	0.01342
Hospitalization [yes/no]	0.50495	0.35714	0	0	1	1	101	99	0.5025	0.48162	0.035481	1.088449	0.675738
Number of days spent in the hospital	8.9596	6.07447	0	0	38	32	101	99	10.344	9.49843	0.045282	1.185969	0.407659

**Table 2:** Basic characteristics of the results obtained in the AIS incl. division by gender.

	All Patients					Men					Women				
	Valid N	Median	Min	Max	SD	Valid N	Median	Min	Max	SD	Valid N	Median	Min	Max	SD
AIS I	200	1.061	0.0	3.0	1.008	101	0.970	0.0	3.0	0.974	99	1.156	0.0	3.0	1.040
AIS II	200	1.360	0.0	3.0	0.740	101	1.287	0.0	3.0	0.712	99	1.438	0.0	3.0	0.765
AIS III	200	0.787	0.0	3.0	0.830	101	0.723	0.0	3.0	0.763	99	0.854	0.0	3.0	0.894
AIS IV	200	0.802	0.0	3.0	0.831	101	0.614	0.0	3.0	0.761	99	1.000	0.0	3.0	0.858
AIS V	200	0.782	0.0	3.0	0.788	101	0.703	0.0	3.0	0.742	99	0.865	0.0	3.0	0.829
AIS VI	200	0.594	0.0	3.0	0.781	101	0.455	0.0	3.0	0.714	99	0.740	0.0	3.0	0.824
AIS VII	200	0.579	0.0	3.0	0.707	101	0.406	0.0	3.0	0.651	99	0.760	0.0	2.0	0.722
AIS VIII	200	1.046	0.0	3.0	0.625	101	0.960	0.0	3.0	0.599	99	1.135	0.0	3.0	0.643
AIS [total]	200	6.895	0.0	24.0	4.667	101	6.099	0.0	24.0	4.337	99	7.707	0.0	22.0	4.870

**Table 3:** Basic characteristics of the results obtained in the AIS divided between hospitalized and non- hospitalized patients.

	Hospitalized					Non-Hospitalized				
	Valid N	Median	Min	Max	SD	Valid N	Median	Min	Max	SD
AIS I	86	1.200	0.0	3.0	1.033	113	0.946	0.0	3.0	0.980
AIS II	86	1.471	0.0	3.0	0.765	113	1.270	0.0	3.0	0.713
AIS III	86	0.953	0.0	3.0	0.962	113	0.658	0.0	2.0	0.694
AIS IV	86	0.765	0.0	3.0	0.882	113	0.838	0.0	3.0	0.792
AIS V	86	0.894	0.0	3.0	0.845	113	0.694	0.0	3.0	0.736
AIS VI	86	0.671	0.0	3.0	0.836	113	0.532	0.0	3.0	0.736
AIS VII	86	0.600	0.0	3.0	0.727	113	0.559	0.0	2.0	0.697
AIS VIII	86	0.882	0.0	3.0	0.606	113	1.171	0.0	3.0	0.616
AIS [total]	86	7.326	0.0	24.0	5.281	113	6.549	0.0	18.0	4.151

**Table 4:** Basic characteristics of the results obtained in the AIS for both hospitalized and non-hospitalized patients divided by gender.

	Hospitalized										Non-Hospitalized									
	Men					Women					Men					Women				
	Valid N	Median	Min	Max	SD	Valid N	Median	Min	Max	SD	Valid N	Median	Min	Max	SD	Valid N	Median	Min	Max	SD
AIS I	51	1.059	0.0	3.0	1.008	35	1.412	0.0	3.0	1.048	50	0.880	0.0	3.0	0.940	63	1.000	0.0	3.0	1.017
AIS II	51	1.353	0.0	3.0	0.770	35	1.647	0.0	3.0	0.734	50	1.220	0.0	2.0	0.648	63	1.311	0.0	3.0	0.765
AIS III	51	0.843	0.0	3.0	0.857	35	1.118	0.0	3.0	1.094	50	0.600	0.0	2.0	0.639	63	0.705	0.0	2.0	0.738
AIS IV	51	0.569	0.0	3.0	0.806	35	1.059	0.0	3.0	0.919	50	0.660	0.0	2.0	0.717	63	0.984	0.0	3.0	0.826
AIS V	51	0.824	0.0	3.0	0.842	35	1.000	0.0	3.0	0.853	50	0.580	0.0	2.0	0.609	63	0.787	0.0	3.0	0.819
AIS VI	51	0.451	0.0	3.0	0.702	35	1.000	0.0	3.0	0.921	50	0.460	0.0	3.0	0.734	63	0.590	0.0	2.0	0.739
AIS VII	51	0.392	0.0	3.0	0.666	35	0.912	0.0	2.0	0.712	50	0.420	0.0	2.0	0.642	63	0.672	0.0	2.0	0.724
AIS VIII	51	0.824	0.0	3.0	0.555	35	0.971	0.0	2.0	0.674	50	1.100	0.0	3.0	0.614	63	1.230	0.0	3.0	0.616
AIS [total]	51	6.275	0.0	24.0	4.924	35	8.857	0.0	22.0	5.478	50	5.920	0.0	16.0	3.686	63	7.048	0.0	18.0	4.452

**Table 5:** Descriptive statistics for the number of symptoms related to COVID-19 by different times of the assessment.

	All Patients					Men					Women				
Number of Symptoms	Valid N	Median	Min	Max	SD	Valid N	Median	Min	Max	SD	Valid N	Median	Min	Max	SD
At present	200	0.704	0.0	5.0	1.192	101	0.614	0.0	5.0	1.058	99	0.796	0.0	5.0	1.316
Acute phase	200	7.141	0.0	16.0	3.145	101	6.693	0.0	16.0	3.158	99	7.602	1.0	15.0	3.079
After 1st week	200	0.015	0.0	3.0	0.213	101	0.000	0.0	0.0	0.000	99	0.031	0.0	3.0	0.303
After 1st month	200	0.513	0.0	9.0	1.251	101	0.446	0.0	7.0	1.100	99	0.582	0.0	9.0	1.392
Acute phase- present	200	8.330	0.0	18.0	3.539	101	7.752	0.0	16.0	3.324	99	8.919	0.0	18.0	3.669

**Table 6:** Correlation of the results obtained in the AIS with the number of symptoms related to COVID-19 by gender (Spearman’s rank-order).

	All Patients				Men				Women			
	At Present	Acute Phase	After 1st Month	Acute Phase- Present	At Present	Acute Phase	After 1st Month	Acute Phase- Present	At Present	Acute Phase	After 1st Month	Acute Phase- Present
AIS I	0.3020	0.0628	0.1086	0.1770	0.1163	0.0820	0.1394	0.1802	0.4666	0.0071	0.0864	0.1606
AIS II	0.2507	0.0779	0.1876	0.2250	0.2324	0.0291	0.1301	0.1514	0.2707	0.0906	0.2470	0.2905
AIS III	0.1609	−0.0146	0.1723	0.0911	0.1102	0.0086	0.0948	0.1037	0.2043	−0.0571	0.2434	0.0805
AIS IV	0.2193	0.1880	0.0589	0.2515	0.1959	0.1662	0.0158	0.2367	0.2352	0.1465	0.0844	0.2083
AIS V	0.2286	0.1306	0.1228	0.2149	0.2350	0.1630	−0.0200	0.2208	0.2187	0.0814	0.2561	0.2105
AIS VI	0.2497	0.2167	0.1327	0.3122	0.1779	0.2035	0.0342	0.2571	0.3078	0.1873	0.2179	0.3384
AIS VII	0.2107	0.2378	0.0903	0.3182	0.0780	0.2153	0.0236	0.2761	0.3136	0.2053	0.1361	0.3055
AIS VIII	−0.0014	0.1008	−0.1266	0.0706	0.0518	0.0741	−0.0739	0.0779	−0.0530	0.0858	−0.1729	0.0274
AIS [total]	0.2771	0.1387	0.1325	0.2605	0.2076	0.1348	0.1168	0.2452	0.3352	0.1069	0.1611	0.2591

**Table 7:** Correlation of the results obtained in the AIS with the number of symptoms related to COVID−19 by hospitalization (Spearman’s rank-order).

	Hospitalized		Non-Hospitalized	
	At Present	Acute Phase	At Present	Acute Phase
AIS I	0.20321	0.07271	0.37848	0.01408
AIS II	0.25194	0.02409	0.22631	0.08627
AIS III	0.06843	−0.04114	0.24284	−0.02104
AIS IV	0.13670	0.19139	0.30123	0.20317
AIS V	0.16015	0.11399	0.27815	0.11154
AIS VI	0.21835	0.18187	0.26744	0.23794
AIS VII	0.13548	0.24889	0.27247	0.22850
AIS VIII	−0.11757	0.03006	0.12733	0.22225
AIS [total]	0.19953	0.11172	0.34245	0.15749

## Data Availability

The study is registered at ClinicalTrials.gov (NCT04 453 748, https://clinicaltrials.gov/ct2/show/NCT04453748 (accessed on 2 August 2022)).
